# Treatment of Sunstroke

**Published:** 1890-09

**Authors:** 


### TREATMENT OF SUNSTROKE.

In a report based on the observation of 60 cases of sunstroke in the New York Hospital, Dr. Swift gives the following account of the attack:

"The patients are suddenly seized, while in the performance of their labors, with pain in the head, and a sense of fullness and oppression in the pit of the stomach, occasionally nausea and vomiting, dimness of vision and insensibility. Surrounding objects appear of a uniform color. In a great majority of cases this was, so far as could be ascertained, blue or purple. In one instance every thing appeared red; in another green, and in another white."

The attack generally occurs during that portion of the day when the heat is of the greatest intensity. It may consist only of a momentary insensibility, the patient evidently fainting, but in severe cases he quickly passes into a state of profound stupor. In the latter, the temperature of the body is notably raised; the skin is burning to the touch, and either dry or moist. The pulse is usually at first slow and full, but becomes rapid and feeble toward the fatal termination. The respirations, more often increased in frequency, are noisy, being accompanied with snoring sounds, sighing or moaning. Muscular spasm and convulsions are of frequent occurrence.

In the management of cases of sunstroke it is important to remember that patients suffering from severe attacks sink very rapidly ; therefore no time should be lost in applying at least the first measures of treatment. Complete rest is of the utmost importance, and, unless the distance is exceedingly short, the removal of the sufferers to their homes, or to hospitals, is attended with great risk, as it may contribute in no small measure to the fatal result.

To apply cold to the head is the first measure to be employed. Ice, if at hand, should be used, if not, a stream of cold water may be poured over the head, back and neck. If the heat of the body is very great, the patient should be immersed in a bath for a considerable time, the water being at first slightly warmed, and then gradually but quickly cooled. If there are no conveniences for bathing, then the body may be freely and persistently sponged with cold water, or the patient can be laid on the floor, covered with a sheet, and, with a sprinkling pot, the cold water may be applied. This douching can be kept up at intervals of a few minutes until the heat of the body is lessened.

If the pulse grows rapid and feeble, then stimulants will be demanded. If the patient is able to swallow, teaspoonful doses of the aromatic spirit of ammonia, well diluted, may be given every half-hour or hour, as the need is apparent. If remedies cannot be administered by the mouth, then brandy by injection is indicated.

In this connection it is well to state that the indiscriminate use of brandy, when patients are overcome by the heat, is extremely pernicious. When the face is blazing and congested, the vessels of the neck stand out and throb, then withhold alcohol. If, however, the pulse is feeble and rapid, then give it freely. So, too, if the skin is cold and clammy, in which condition collapse is indicated, cold applications must not be employed, but heat should be applied instead, and the patient briskly rubbed with hot flannels to restore circulation.

The commencing treatment herein described will be appropriate in all cases. With a knowledge of the measures of relief, they can be applied at once and be persisted in until a physician arrives, and assumes the care of the unfortunate.

